# Lymphocytic infiltration in the cutaneous lymphoma microenvironment after injection of TG1042

**DOI:** 10.1186/1479-5876-11-226

**Published:** 2013-09-25

**Authors:** Nathalie Accart, Mirjana Urosevic-Maiwald, Reinhard Dummer, Vincent Bataille, Nadine Kehrer, Cristina Niculescu, Jean-Marc Limacher, Marie-Pierre Chenard, Jean-Yves Bonnefoy, Ronald Rooke

**Affiliations:** 1 Transgene S.A., Boulevard Gonthier d’Andernach, Parc d’Innovation, 67405 Cedex Illkirch Graffenstaden, France; 2 C.H.U. Hautepierre, Laboratoire d’Anatomie Pathologique, 67000 Strasbourg, France; 3 Department of Dermatology, University Hospital of Zurich, Gloriastrasse 31, CH-8091 Zürich, Switzerland; 4 ElsaLysbiotech, 650 Boulevard Gonthier d'Andernach, Parc d'Innovation, F-67400 Illkirch Graffenstaden, France

**Keywords:** Cutaneous lymphoma, TG1042, Lymphocytes, Cytotoxicity

## Abstract

**Background:**

Primary cutaneous lymphomas (CLs), characterized by an accumulation of clonal T or B lymphocytes preferentially localized in the skin, have been successfully treated with interferons (IFNs) which counterbalance the Th2-immunosuppressive state associated with this pathology. In a phase I/II clinical trial, we correlated the local immune infiltrate and the anti-tumor effects of repeated intralesional administrations of an adenovirus vector expressing human interferon-gamma (IFN-g) termed TG1042, in patients with advanced primary cutaneous T-cell lymphomas (CTCL) or multilesional cutaneous B-cell lymphomas (CBCL).

**Methods:**

For each patient, variation in time of specific lymphocyte populations, defined by immunohistochemical stainings, was assessed in biopsies of injected lesions. For each patient, the change in local immune response was associated with the patient’s objective response at the end of the study.

**Results:**

Immunohistochemical analyses of biopsies indicate that infiltration of CD8^+^ T lymphocytes and of TIA-1^+^ cytotoxic T-cells in lesions injected with TG1042 correlates with clinical benefit.

**Conclusions:**

These data suggest for the first time that a CD8^+^ cytotoxic infiltrate, induced by local expression of IFN-g correlates with a clinical response.

**Trial registration:**

The phase I step (TG1042.01) does not have a registration number. The phase II step (TG1042.06) registration number was NCT00394693.

## Background

Cutaneous lymphoma (CL), with an incidence of 1 case per 100,000 individuals per year, is a group of at least fifteen rare diseases [1]. CL span a wide array of clinical aspects and histological subtypes which correspond to clonal T or B cells, characterized by their expression of lymphoid cell markers, homing and migrating mainly in the skin [2]. In CTCL, the CD4/CD8 ratio has been associated with the tumor stage of the disease [3]. The granulomatous slack skin is an extremely rare subtype of MF characterized by epidermal focal infiltration of small atypical T cells with a CD3^+^, CD4^+^, CD8^-^ phenotype [4]. Large T-cell CD30^+^ lymphoma (ALCL) demonstrate diffuse infiltrates of large anaplastic CD30^+^ T cells which are CD4^+^ with variable loss of the pan-T-cell antigen CD3 [5], [6]. The atypical cells in the lymphomatoid papulosis types A and C lesions have the same phenotype as the tumor cells in ALCL. The type B lesions have a CD3^+^, CD4^+^, CD8^-^, CD30^-^ phenotype [4]. Pleomorphic small/medium-sized T-cell lymphoma is characterized by atypical lymphoid cells expressing the CD4 molecule [5,7]. B-cell lymphomas are characterized by a dense infiltrate of tumor cells expressing B cell-specific surface antigens such as CD20 and CD79α [8,9]. In summary, the CD4 marker is characteristic of cutaneous T-cell lymphomas (CTCL) and the CD20 and CD79α markers are features of cutaneous B-cell lymphomas (CBCL).

The body of information now encompassing this family of diseases has motivated the development and justified the use of numerous treatment modalities such as kinase, methylation or histone acetylation inhibitors, mAbs, rexinoids and photopheresis [10]. Disease progression is associated with a decrease in the production of Th1 cytokines and a shift towards the anti-inflammatory Th2 phenotype and some of the successful treatments have been shown to restore the Th1/Th2 imbalance [11]. Moreover, this group of diseases has been successfully treated with interferons and cytokines that counterbalance the Th2-skewing [12-14].

IFN-g is the principal cytokine associated with the Th1 phenotype. It acts in a positive feedback loop as it is produced by activated Th1 cells and stimulates differentiation of Th0 cells into Th1 cells while suppressing Th2 differentiation (reviewed in [15]). However, using IFN-g to treat pathologies resulting from a Th1/Th2 imbalance is hampered by the significant side effects associated with systemic administration of recombinant cytokines along with their short half-life. This has led to the development of alternative delivery systems such as TG1042, a non-replicating adenovirus type 5 vector containing a human IFN-g cDNA insert under the control of the cytomegalovirus promoter [16]. Intralesional delivery of human IFN-g-expressing adenovirus allows reaching high local concentrations of this cytokine, as shown by quantitative RT-PCR in 7 out of 9 patients following the first treatment cycle. The concentrations reached induce lymphocytic infiltration and activation of local effector cells [16]. We have developed and used TG1042 in clinical trials in CLs [16,17]. The work presented here describes the changes in the infiltrates throughout treatment, consisting in repeated intratumoral injections of TG1042, in patients that participated in these phase I/II clinical trials using extensive immunohistochemistry phenotyping. Our results show that patients whom responded favourably after intralesional injections of TG1042 had an increase in the frequency of CD8^+^ cells and T-cell-restricted intracellular antigen (TIA-1 or cytotoxic granule-associated RNA binding protein) positive cytotoxic cells relative to baseline levels. These results are indicative that the immune reaction is a critical component in the antitumor response in lymphoma.

## Materials and methods

Thirty patients with advanced primary CTCL or multilesional CBCL were enrolled in the phase I step (cohort 4 – Patients 10 to 18) and in the phase II step (cohort 5 – Patients 19 to 39) of the clinical studies supported by Transgene S.A. (Strasbourg France). The study was approved by the local institutional ethical committees and the national authorities for biosafety in Switzerland, Germany, and France, and conducted in accordance with the ethical principles of the Declaration of Helsinki, in compliance with the approved protocol, and its amendments, and good clinical practices. Patients received repeated intratumoral administration of TG1042 (3E + 11 viral particles (vp) per injection, divided in up to three lesions). Among these 30 patients, 20 were evaluable for efficacy. In this latter population, 19 patients were assessed for the nature of the lymphocytic infiltrate in skin biopsies by immunohistochemistry at different time points throughout the study (for Phase I and Phase II details, refer to [16,17]). Details of each patient in the study population, including histological type and stage are given in Table 1. In both cohorts, patients received intratumoral injections of TG1042 in the same lesions on days 1, 8 and 15 of each cycle (for details please refer to [16,17]). Patients did not receive treatment the fourth week. Each cycle lasted 4 weeks and response to treatment was evaluated by measuring tumor surfaces at baseline and at the end of each cycle. The local response was divided in four categories: Complete Response (CR) was defined as the absence of detectable residual disease; Partial Response (PR) was defined as a greater than 50% decrease in the size of pre-existing lesions or if greater than 50% nodular or plaque-like lesions became macules without evidence of internal involvement; Stable Disease (SD) was defined as any response that did not meet the criteria of complete response, partial response or Progressive Disease (PD); the latter was defined as the appearance of new lesions, an increase of 25% in the size of previously existing lesions, a change from macular to plaque-like or nodular lesions in more than 25% of previously existing lesions or any evidence for developing internal manifestation. Patients showing CR or PR are considered as responders while patients showing SD or PD are considered as non-responders. In absence of progressive disease, patients received another cycle with a maximum of 12 cycles. Therapy was stopped when disease progressed or when toxicity was noticed. Biopsies were collected at Baseline (between days −14 and 0 relative to initiation of the treatment), 24 hours after the third injection of a cycle (noted Cx where x corresponds to the cycle number) or at the last visit (noted Last Visit) corresponding to at least 4 weeks after last TG1042 injection. When a CR was observed, treatment was stopped and a biopsy was taken (noted Cx). Table 1 summarizes the time of biopsies sampling for each patient.

**Table 1:** Patient’s characteristics

**Patient #**	**Subtype**^**1**^	**Stage at baseline**	**Biopsies**^**2**^
**10**	FCBCL	NA	Baseline, C1
**13**	CD30^+^ ALCL	IIB	Baseline, C1
**16**	CD30^+^ ALCL	IIA	Baseline, C1, C2
**17**	GSS	IB	Baseline, C1, C2, C3, C4, C6, C9, C10
**18**	MF	IIB	Baseline, C1, C2
**20**	CD30^+^ ALCL	IIB	Baseline, C1
**24**	Pleomorphic CTCL	IB	Baseline, C1, C4, Last Visit
**25**	MF	IB	Baseline, C1, C4, Last Visit
**27**	MF	IIB	Baseline, C1
**29**	MF	IB	Baseline, C1, Last Visit
**30**	MF	IB	Baseline, C1, C4
**31**	MF	IIB	Baseline, C1, C4
**33**	DLBCL	T3a	Baseline, C1
**34**	MF	IB	Baseline, C1, Last Visit
**35**	MZBCL	T3b	Baseline, C1
**36**	MF	IB	Baseline, C1, C4
**37**	CD30^+^ ALCL	IIB	Baseline, C2
**38**	MF	IB	Baseline, C1, C4, Last Visit
**39**	MF	IIB	Baseline, C1, C3

Summary of relevant information for each patient analysed (patient number, histology subtype, stage at baseline and time of biopsies sampling).

NA not applicable.

^1^Pathology subtypes: ALCL indicates anaplastic large cell lymphoma, MF Mycosis fungoides, CBCL cutaneous B-cell lymphoma, DLBCL diffuse large B-cell lymphoma, FCBCL follicle center B-cell lymphoma, MZBCL marginal zone B-cell lymphoma, Pleomorphic CTCL Pleomorphic cutaneous T-cell lymphoma, LyP lymphomatoid papulosis, GSS granulomatous slack skin.

^2^Cx: C:Cycle. x: cycle number at which the examined biopsies were sampled. See Material and Methods for Cycle definition.

Biopsies, corresponding to injection sites, were fixed in formalin, embedded in paraffin and sectioned. Briefly, formalin-fixed, paraffin embedded tissue sections were deparaffinized in xylene and rehydrated in an ethanol/water gradient. After epitope retrieval with antigen-specific methods, endogenous peroxidases were inactivated and non-specific staining was blocked by saturating sections with 10% normal goat serum. Marker-specific primary antibodies and their conditions of use are described in Table 2. Sections were incubated for one and a half hour at room temperature with primary antibodies diluted as indicated in Table 2 in PBS or TBS. Revelation steps were adapted for each primary antibody and applied for 30 minutes (Table 2). The horseradish peroxydase activity was visualized by diaminobenzidine (DAB) precipitation (Dako). The revelation was standardized for each marker. Slides were counterstained with hematoxylin and mounted in Eukitt® (Labonord) after dehydration.

**Table 2:** Antibodies used for immunohistochemical evaluation

**Antibody**	**Expression**	**Clone**	**Isotype**	**Reference**	**Source**	**Antigen retrieval**	**Dilution**	**Amplification system**
**CD3**	T-lymphocytes		rabbit pc IgG	A0452	DAKO	EDTA buffer pH9 (Target Retrieval®, Dako)	1/100	0.5% dilution of horse biotinylated secondary antibody + ABC amplification system, Vector Laboratories
						45 minutes - 95°C		
**CD4**	helper and regulatory T-lymphocytes	1 F6	mouse mc IgG1	NCL-CD4-1 F6	NOVOCASTRA	10 mM Citrate Buffer pH6	1/20	non diluted anti-mouse Envision® system, Dako
		4B12	mouse mc IgG1 κ	NCL-CD4-368	NOVOCASTRA	3 x 5 minutes – 750 W	1/20	non diluted anti-mouse Envision® system, Dako
**CD8**	cytotoxic T-lymphocytes	C8/144B	mouse mc IgG1κ	#M7103	DAKO	EDTA buffer pH9 (Target Retrieval®, Dako)	1/50	non diluted anti-mouse Envision® system, Dako
						45 minutes - 95°C		
**CD20cy**	mature and immature B-lymphocytes	L26	mouse mc IgG2a κ	M7055	DAKO	10 mM Citrate Buffer pH6	1/200	0.5% dilution of horse biotinylated secondary antibody + ABC amplification system, Vector Laboratories
						2 minutes - 750 W + 7 minutes -250 W		
**CD56**	Natural Killer cells	564	mouse mc IgG2b	NCL-CD56-504	NOVOCASTRA	10 mM Citrate Buffer pH6	1/50	non diluted anti-mouse Envision® system, Dako
						45 minutes – 95°C		
**CD79αcy**	B cells from early pre-B to plasma cell stage and neoplastic B-lymphocytes	HM57	mouse mc IgG1κ	M7051	DAKO	EDTA buffer pH9 (Target Retrieval®, Dako)	1/50	0.5% dilution of horse biotinylated secondary antibody + ABC amplification system, Vector Laboratories
						45 minutes - 95°C		
**TIA-1**	CD4 and CD8 positive T cells, granulocytes, monocytes and activated NK cells	2G9	mouse mc IgG1	PN IM2550	IMMUNOTECH	10 mM Citrate Buffer pH6	1/50	non diluted anti-mouse Envision® system, Dako
						2 minutes - 750 W + 7 minutes -250 W		
**MHC Class I**	most nucleated cells	HC10	mouse monoclonal IgG2a	SB03 546	kindly provided by Dr S. Ferrone, G. Gaslini Institute, Italy	EDTA buffer pH9 (Target Retrieval®, Dako)	1/20	non diluted anti-mouse Envision® system, Dako
						45 minutes - 95°C.		

MHC Class I: Major Histocompatibility Complex I (Human Leukocyte Antigen A, B, C); mc: monoclonal; pc: polyclonal; TIA-1: T-cell restricted intracellular antigen-1.

**Figure 1:**
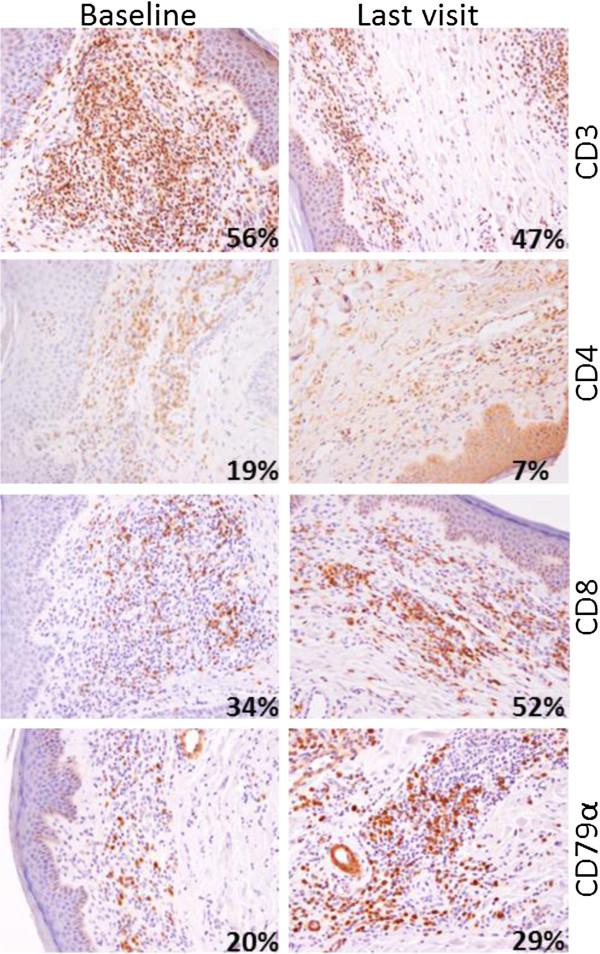
**Immune infiltrates in a CTCL complete responder before and after TG1042 treatment.** Patient #24 at baseline (left panels) and at last visit, 4 weeks after last TG1042 injection (right panels). Percentages on pictures correspond to the ratio of DAB-positive cells on total hematoxylin-stained nuclei in the whole infiltrating population, as determined by ImageJ software macro-commands. The proportion of CD3 and CD4 expressing cells decreased at the last timepoint; the number of CD8 and CD79 α expressing cells increased between baseline and last visit.

**Figure 2:**
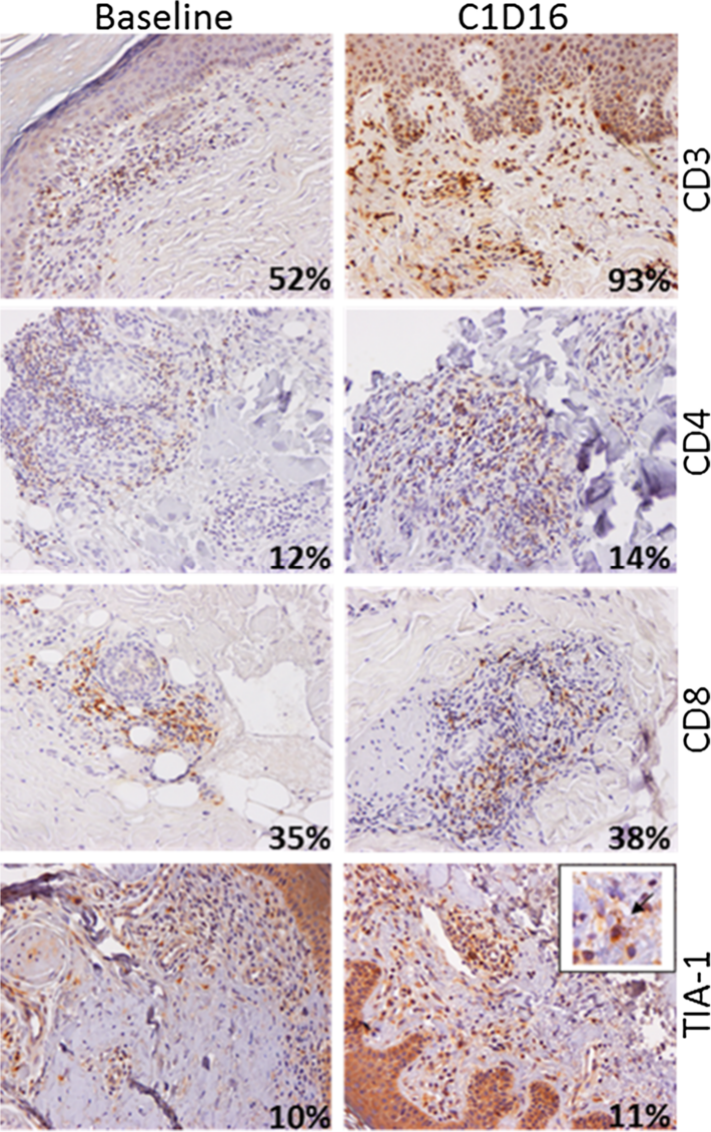
**Immune infiltrates in a CTCL progressive disease before and after TG1042 injections.** Patient #27 at baseline (left panels) and at C1D16 (right panels). Percentages on pictures correspond to the ratio of DAB-positive cells on total hematoxylin-stained nuclei in the whole infiltrating population, as determined by ImageJ software macro-commands. The frequency of CD3 expressing cells increased between baseline and C1D16; CD4, CD8 and TIA-1 were stable markers. (Insert) TIA-1 staining is associated with intracellular granules.

**Figure 3:**
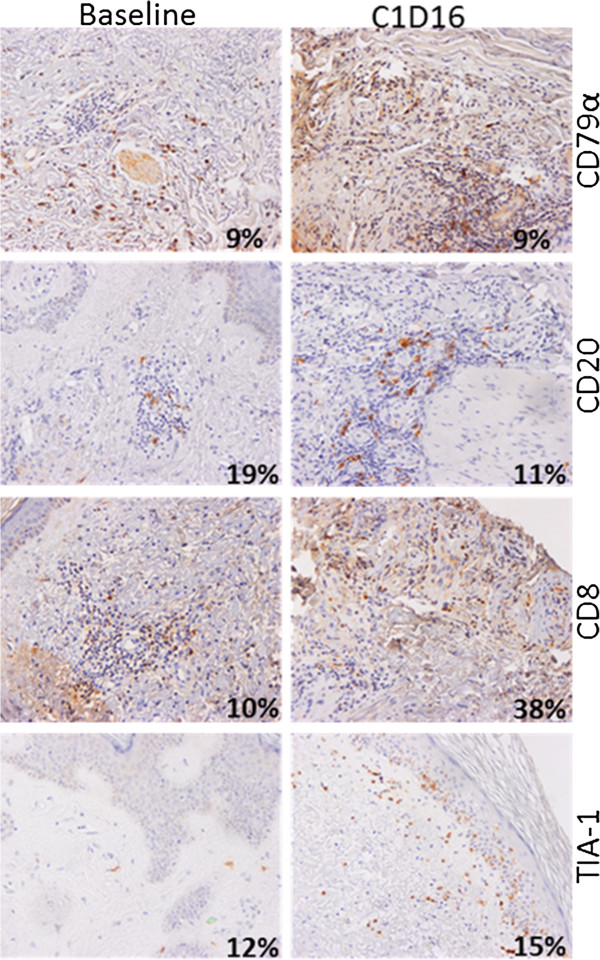
**Immune infiltrates in a CBCL complete responder before and after TG1042 injections.** Patient #33 at baseline (left panels) and at C1D16 (right panels). Percentages on pictures correspond to the ratio of DAB-positive cells on total hematoxylin-stained nuclei in the whole infiltrating population, as determined by ImageJ software macro-commands. CD79α, CD20 and TIA-1 are stable markers; the frequency of CD8 expressing cells increased between baseline and C1D16.

**Figure 4:**
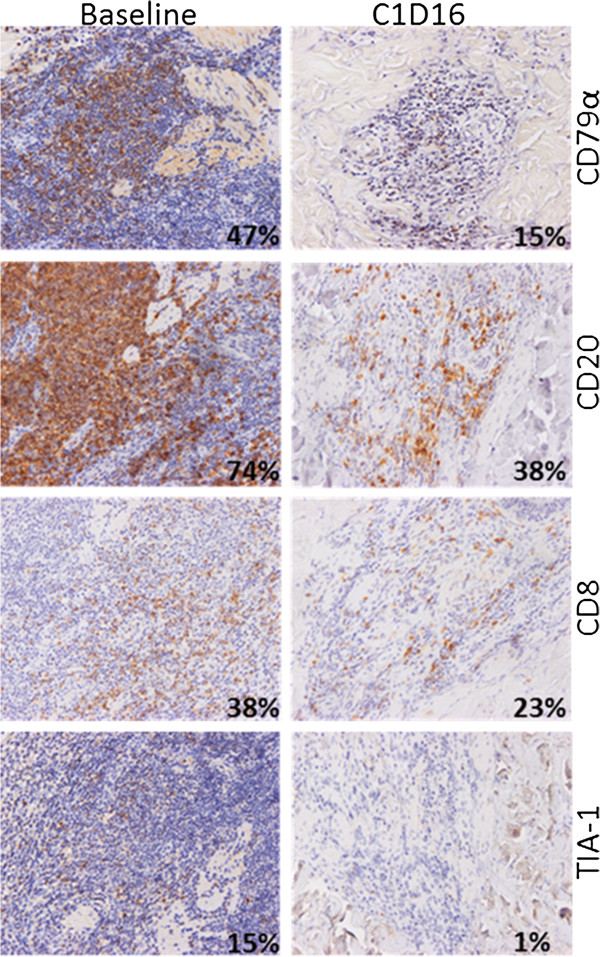
**Immune infiltrates in a CBCL partial responder before and after TG1042 injections.** Patient #35 at baseline (left panels) and at C1D16 (right panels). Percentages on pictures correspond to the ratio of DAB-positive cells on total hematoxylin-stained nuclei in the whole infiltrating population, as determined by ImageJ software macro-commands. The frequency of CD79α, CD20, CD8 or TIA-1 expressing cells decreased between baseline and C1D16.

Staining was visualized using a Nikon Eclipse E800 microscope with 10x and 40x objectives and images were acquired with a Nikon DXM1200 camera and the NIS-Elements AR version 2.30 software (Nikon). For each patient and for each marker except MHC Class I, variations in the number of infiltrated lymphocytes revealed by specific antibodies were first estimated by two independent histologists (N.A. and N.M.). Since MHC Class I is present on most cell types, we resorted to intensity variation estimates by visual appreciation. Results were tabulated to estimate the evolution in time of each marker for one patient. Four categories have been defined depending on the increased, stable, decreased or absence of stained cell between baseline and the last biopsy (Additional file 1: Table S1). The proportions of positive cells were confirmed on four patients, representative of various responses, using in house-developed, ImageJ free software (http://rsb.info.nih.gov/ij) macro-commands on entire section scans (Nanozoomer, Hamamatsu). Commands quantify objects corresponding to DAB-positive cells and hematoxylin-stained nuclei present in the whole infiltrating population. The results are given as percentage of positive cells on total nuclei (Figures 1, 2, 3 and 4).

## Results

We analyzed the nineteen patients at the indicated time-points and estimated the levels of expression of CD3, CD4, CD8, CD20cy, CD79αcy, MHC Class I and TIA-1. Additional file 1: Table S1 compiles the results.

Three of the four CTCL patients with a complete response (CR) were CD30^+^ ALCL (#13, #16, and #20). The pleomorphic CTCL patient (#24) also had a CR for which the immune infiltrate is shown in Figure 1. These patients showed an increase in the proportion of CD8^+^ cells between baseline and the last time point. The cytotoxic marker TIA-1 was increased or stable. The CD4^+^ lymphocytes, characteristic of CTCL, were either stable or decreased in number.

Of the three evaluable MF CTCL partial responders (PR) (#25, #31, and #36), one showed increase in the cellular infiltrate while it remained unchanged for the two other patients. All cases presented an increased proportion of CD8^+^ lymphocytes in the biopsy and two patients out of three showed an increased numbers of TIA-1^+^ and CD79α^+^ lymphocytes between baseline and the last time point analyzed. Likewise, two patients out of three showed increased numbers of CD3^+^ and CD4^+^ lymphocytes.

The six evaluable CTCL patients (#18 CD30^+^ ALCL, #29 MF, #38 MF, #39 MF, #17 GSS and #37 CD30^+^ ALCL) with a stable disease (SD) had a stable or decreased frequency of CD3^+^ cells. Four out of six patients had an increased frequency of CD8^+^ cells between baseline and the last time point examined. The others markers did not vary in a consistent way.

The three MF CTCL patients (#27 (Figure 2), #30, and #34) with a progressive disease (PD) showed stable numbers of CD8^+^ lymphocytes (2/3) while the proportion of CD3^+^ (3/3) or CD4^+^ lymphocytes (2/3) increased. One patient showed stable numbers of TIA-1^+^ or CD79α^+^ lymphocytes, one patient showed a decreased number of TIA-1^+^ cells and a stable number of CD79α^+^ lymphocytes, and one patient showed increased numbers of TIA-1^+^ or CD79α^+^ lymphocytes.

MHC Class I was evaluated using an intensity criterion instead of the number of positive cells. One out of the seven evaluable CTCL responders (CR + PR) had increased intensity of MHC Class I staining while the six others were stable. Five out of the nine evaluable CTCL non responders (SD + PD) had stable intensity of staining; the others had increased (2/9) or decreased intensity (2/9).

The CBCL complete responders (#10 FCBCL and #33 DLBCL) showed an increased number of CD3^+^ and of CD8^+^ lymphocytes between baseline and C1 associated with an unchanged aspect of cellular infiltrate for patient #33 (Figure 3). The cytotoxic marker TIA-1 was stable or increased and the number of CD4^+^ cells was stable or decreased. The MHC Class I staining intensity was stable or increased.

The MZBCL partial responder (#35, Figure 4) showed a smaller cellular infiltrate associated to a decreased number of CD4^+^ and CD8^+^ lymphocytes between baseline and C1D16. The MHC Class I staining intensity was stable.

In both type of responders, we observed a stable or decreased number of CD20^+^ cells and CD79α^+^ cells, characteristic of the pathology.

## Discussion

Cutaneous lymphomas are neoplasms from the skin-associated lymphoid tissue. Skin infiltrates of clonal CD3 positive, CD4 positive cells is characteristic of most cutaneous T-cell lymphomas (CTCL), whereas increased numbers of CD20 and CD79α positive cells in the skin are features of cutaneous B-cell lymphomas (CBCL) [4].

CL depend on the presence of dominant Th2-type cytokines in their microenvironment and immunotherapies using IFN-a, IFN-g or IL-12 have antitumor activity [11,18]. Mechanistically, IFN-g inhibits production of Th2-cytokines by lymphoma cells *in vitro*[19]. It is also required for IL-12 secretion by antigen-presenting cells which pushes the immune response towards the Th1 phenotype [20]. Moreover, the IFN-g induced up-regulation of expression of the major histocompatibility complex molecules increases the susceptibility of tumor cells to be recognized by the host’s immune system [21]. Given these arguments, cutaneous lymphoma represents a good potential target for adenovirus-mediated IFN-g gene delivery.

We analyzed the local immune infiltrates in injected lesions obtained from advanced primary CTCL and multi-lesional CBCL patients enrolled in the Phase I and II steps of a clinical trial (Phase I/II study TG1042.01). Patients received repeated intralesional injections of TG1042 and we estimated the changes in the lymphocyte populations at various timepoints throughout the study. We analyzed the CD4 marker as characteristic of CTCL and the CD20 and CD79α markers as features of CBCL. The other markers used in this study more generally define lymphocytic infiltration associated with an immune response: CD3 for the complete T lymphocyte content, the CD8 marker is characteristic of effector T cells and the cytotoxicity marker protein TIA-1 contained in the granules involved in lysis [22]. We also looked at MHC Class I expression since it is known to be induced by IFN-g and is the target for cytotoxic T cells [21].

In the Per Protocol population of twenty evaluable patients, only nineteen patients were evaluated in this immunological study. Local clinical responses (Complete and Partial Response) were observed in ten out of nineteen (53%) patients. Four out of seven (57%) responding CTCL patients had stage IB or IIA disease at baseline while five out of nine (55%) non-responders (Stable and Progressive Disease) had stage IB disease at inclusion. Although the limited number of patients included in this work would not allow statistical analysis, the equal distribution between responders and non-responders tends to show that there is no association between the stage of disease at baseline and the clinical outcome following the treatment. This observation is not applicable to CBCL patients because all patients are complete responders.

It is interesting to note that for the CTCL from the cohort 5, the histopathologist from the central laboratory (Dr Marie-Pierre Chenard at CHU Hautepierre, Strasbourg, France) described the variation in size of the lymphocytic infiltration either as increasing, unchanged or decreasing and correlated the size of the infiltrates with disease progression. Without knowing the nature of the infiltrating cells, the pathologist described the responders as “increased” or “unchanged” and the non- responders as “unchanged” or “decreased” thereby associating complete responses with important lymphocytic infiltration (data not shown). This latter observation is in good agreement with what we observed in the cohort of sixteen CTCL patients studied here. For all the seven CTCL responders, injection of TG1042 was associated with an increase in CD8^+^ cells (100%). Five out of these seven patients had increased numbers of TIA-1^+^ cells and one patient had an increased intensity of MHC Class I from baseline (11%). Five out of the nine CTCL non-responders have an increased number of CD8^+^ cells (55%), four out of these nine patients have a stable number of CD8^+^ cells or no CD8^+^ cells, five out of nine had increased number of TIA-1^+^ cells (56%) and two out of nine have an increased intensity of MHC Class I staining (22%). Similar results were seen in regressing CTCL tumors after intralesional administration of IL-12 [14] or after injection with adenovirus-engineered IL-2 expressing autologous plasma cells [22]. Moreover, the CTCL complete response correlate with a decrease in number of CD4^+^ cells reflecting the disappearance of lesions. Surprisingly, although we expected IFN-g-mediated enhanced MHC Class I expression, only three out of sixteen CTCL showed an increased intensity of the MHC Class I staining and no correlation with clinical responses. These results suggest that IFN-g promotes the development of cytotoxic CD8^+^ T cells-mediated immune responses directed against the lymphoma cells [23,24].

## Conclusion

In summary, intralesional injections of TG1042 (Adenovirus-IFN-g) induce changes in the nature of the infiltrate differing from the initial lymphoma. CBCL and CTCL patients who responded had an increase in frequency of CD8^+^ cells and TIA-1^+^ cells, indicative of an immune response which is a critical component in the antitumor response in lymphoma.

## Consent

Prior to entering the study, patients were fully informed orally and in writing about the objectives and implications of the study. They were informed that the data disclosed in the reports and publications resulting from the study would systematically be treated in an anonymous manner, i.e. without identity revelation. All patients provided written informed consent.

## Abbreviations

Ad: Adenovirus; ALCL: Anaplastic large cell lymphoma; CBCL: Cutaneous B cell lymphoma; CL: Cutaneous lymphoma; CR: Complete response; CTCL: Cutaneous T cell lymphoma; DLBCL: Diffuse large B-cell lymphoma; DNA: Deoxyribonucleic acid; FCBCL: Follicle center B-cell lymphoma; GSS: Granulomatous slack skin; IFN: Interferon; IFN-a: Interferon alpha; IFN-g: Interferon gamma; LyP: Lymphomatoid papulosis; MF: Mycosis fungoides; MZBCL: Marginal zone B-cell lymphoma; NA: Not applicable; PD: Progressive disease; Pleomorphic CTCL: Pleomorphic cutaneous T-cell lymphoma; PR: Partial response; SD: Stable disease.

## Competing interests

This work was supported by Transgene SA. The authors declare that they have no competing interests.

## Authors’ contributions

MUM and RD were involved in the clinical study, its design and coordination, and helped to comment the manuscript, VB and JML conceived of the study, and participated in its design and coordination and helped to draft the manuscript, CN contributed to the final manuscript as project manager, MPC was involved as expert for the anatomical description of the lymphocytic infiltration, NK carried out all the immunohistochemical stainings and their first interpretation, NA carried out the second interpretation of the stainings, the design of the ImageJ macro-command for the quantification of the stainings and drafted the manuscript, RR drafted the manuscript, and JYB approved the final version of the manuscript. All authors read and approved the final manuscript.

## Supplementary Material

Additional file 1: Table S1 Summary of immunostaining results. Click here for file
