# Supplementary material for: Lymphocytic infiltration in the cutaneous lymphoma microenvironment after injection of TG1042
Source: J Transl Med. 2013 Sep 25;11:226. doi: 10.1186/1479-5876-11-226 (PMC4015780; doi:10.1186/1479-5876-11-226)
Supplement: Additional file 1: Table S1 — Summary of immunostaining results. [file 1479-5876-11-226-S1.docx]

**Table 3. Summary of immunostaining results.**

| **Patient No.** | **Subtype** | **Stage** | **Local response** | **CD8** | **TIA-1** | **CD3** | **CD4** | **MHC**  **Class I** | **CD20** | **CD79α** |
| --- | --- | --- | --- | --- | --- | --- | --- | --- | --- | --- |
| 10 | FCBCL | NA | **CR** |  |  |  |  |  |  |  |
| 33 | DLBCL | T3a | **CR** |  |  |  |  |  |  |  |
| 35 | MZBCL | T3b | **PR** |  |  |  |  |  |  |  |
| 13 | CD30+ ALCL | IIb | **CR** |  |  |  |  |  |  |  |
| 16 | CD30+ ALCL | IIa | **CR** |  |  |  |  |  |  |  |
| 20 | CD30+ ALCL | IIb | **CR** |  |  |  |  |  |  |  |
| 24 | Pleomorphic CTCL | Ib | **CR** |  |  |  |  |  |  |  |
| 25 | MF | Ib | **PR** |  |  |  |  |  |  |  |
| 31 | MF | IIb | **PR** |  |  |  |  |  |  |  |
| 36 | MF | Ib | **PR** |  |  |  |  |  |  |  |
| 17 | GSS | Ib | **SD** |  |  |  |  |  |  |  |
| 18 | MF | IIb | **SD** |  |  |  |  |  |  |  |
| 29 | MF | Ib | **SD** |  |  |  |  |  |  |  |
| 38 | MF | Ib | **SD** |  |  |  |  |  |  |  |
| 39 | MF | IIb | **SD** |  |  |  |  |  |  |  |
| 37 | CD30+ ALCL | IIb | **SD** |  |  |  |  |  |  |  |
| 30 | MF | Ib | **PD** |  |  |  |  |  |  |  |
| 34 | MF | Ib | **PD** |  |  |  |  |  |  |  |
| 27 | MF | IIb | **PD** |  |  |  |  |  |  |  |

This table summarizes information relative to each patient classified by pathology type. It includes, the pathology subtype with associated stage the patient identification, the local response at the last visit and the changes in the markers studied.

**Legend**

|  | **increased** |  | **stable** |  | **decreased** |  | **negative** |
| --- | --- | --- | --- | --- | --- | --- | --- |

CR: Complete Response

PR: Partial Response

SD: Stable Disease

PD: Progressive Disease

NA not applicable

NA not applicable

^1^ Pathology subtypes: ALCL indicates anaplastic large cell lymphoma, MF Mycosis fungoides, CBCL cutaneous B-cell lymphoma, DLBCL diffuse large B-cell lymphoma, FCBCL follicle center B-cell lymphoma, MZBCL marginal zone B-cell lymphoma, Pleomorphic CTCL Pleomorphic cutaneous T-cell lymphoma, LyP lymphomatoid papulosis, GSS granulomatous slack skin.
